# The Impact of Economic Policy Uncertainty on Regional Total-Factor Energy Productivity in China

**DOI:** 10.3390/ijerph20042855

**Published:** 2023-02-06

**Authors:** Bin Wang, Chenyang Meng, Hongwei Yu

**Affiliations:** 1 School of Economics, Wuhan Textile University, Wuhan 430200, China; 2 School of Economics, Huazhong University of Science and Technology, Wuhan 430074, China; 3 Institute of Quality Development Strategy, Wuhan University, Wuhan 430072, China

**Keywords:** economic policy uncertainty, total-factor energy productivity, energy market, government intervention

## Abstract

This paper investigates the effect and possible mechanism of implicit macroeconomic policy uncertainty on regional energy productivity in China. This study takes the unexpected output of environmental pollution from energy consumption into account and uses the DEA-SBM method to measure the regional total-factor energy productivity (RTFEP) of prefecture cities in China from 2003 to 2017. Based on the economic policy uncertainty (EPU) index constructed by Baker et al. this paper estimates the effects of EPU on RTFEP and finds that there is a significant negative relationship between them. Specifically, for every unit increase in EPU, RTFEP will decrease by 5.7%. This paper further examines the mechanism of EPU on RTFEP from the perspective of the market and the government and finds that EPU exerts a restraining effect on RTFEP by affecting the consumption structure of the energy market and government intervention in the economy. In addition, the results show that there is heterogeneity in the impact of EPU on RTFEP which varies between resource-based cities at different development stages and different dominant resources. Finally, this paper proposes to cope with the negative impact of EPU on RTFEP by optimizing the energy consumption structure, regulating government investment areas, and transforming the economic development mode.

## 1. Introduction

Energy is considered as an essential production factor for economic growth. Energy productivity is generally understood as the energy consumption under a given economic output condition, and it is an important manifestation of a more intensive economic growth mode [1,2]. The existing literature has carried out a lot of research on energy productivity, mainly focusing on technology, structure, system, and other aspects, to determine the factors affecting the improvement of regional energy productivity and to provide a positive strategy for the transformation of the economic growth mode to an intensive one.

Regional energy productivity is closely related to the economic growth mode, so the study of energy productivity is inseparable from an understanding of the overall macroeconomic policy. In particular, the frequent occurrence of various black swan incidents in recent years, such as China–US trade frictions, Brexit, 2019-nCoV, and the crash of US stocks, have caused global economic uncertainty to increase dramatically. According to the global economic policy uncertainty index calculated by Davis et al. [3], the global economic policy uncertainty index in 2019 has reached twice the level of that in 2008. Facing the increasing uncertainty of global economic policies, governments of various countries have issued more frequent economic policies to respond to the impact of uncertainty by changing the mode of economic growth. Under this circumstance, it is necessary to analyze the changes in regional energy productivity, which is an important indicator of economic growth.

This paper focuses on economic policy uncertainty and regional energy productivity. First of all, in order to make energy productivity better reflect the intensive growth mode of the regional economy, a “total-factor” measurement of energy productivity was conducted. Compared with the traditional single-factor method, the calculation of total-factor energy productivity further considers the impact of capital and labor input. At the same time, pollutants are included as undesirable outputs in the slack-based measure (SBM), which makes the measurement result closer to the green energy productivity in the context of sustainable development.

Second, this paper uses the economic policy uncertainty index developed by Becker et al. to estimate the effect of EPU on the total-factor energy productivity of Chinese cities. The study finds that for every unit increase in EPU, the RTFEP will decrease by 5.7%. This result remains stable after excluding city samples of different grades. Finally, the mechanism by which EPU affects RTFEP was examined. Studies have shown that EPU has a negative impact on energy productivity through energy market adjustment and local government intervention.

Compared with the existing research, this paper mainly contributes in the following three ways. The first is to expand the understanding of implicit macroeconomic factors that affect regional energy productivity. The existing research on the influencing factors of energy productivity mainly focuses on the following three aspects. First, technical factors, including technological progress [4], R&D investment [5], and human capital [6]; second, structural factors, including industrial structure [7], energy consumption structure [8], and ownership structure [9]; and third, institutional factors, including openness [10], marketization [11], and environmental regulation [12].

These are all explicit economic factors that affect energy productivity. This paper proves that under the background of increasing global economic policy uncertainty, in addition to the influence of explicit factors, implicit economic policy uncertainty is also a part that cannot be ignored in the process of economic operation. On the one hand, the uncertainty of the implicit economic policy will lead to the rise in energy prices, which will change the energy consumption structure to a high energy consumption of coal, thus reducing regional energy productivity. On the other hand, in the face of uncertain economic policy, the government will issue a series of loose fiscal policies to stimulate economic growth while also exacerbating the problem of overcapacity, especially when a large amount of investment has poured into infrastructure construction industries such as steel and cement, which causes a reduction in energy productivity brought about by the intervention of government. Meanwhile, the increase in energy consumption and environmental pollution caused by the political tournament system of local officials with GDP growth as the core will further reduce regional energy productivity.

The second is to further enrich the understanding of the negative impact of EPU. The existing literature has conducted research on a series of negative effects of EPU on macroeconomics and microenterprises, including a decline in GDP, investment, consumption, and exports [13,14]; exacerbating RMB exchange rate fluctuation [15]; inhibiting corporate investment [16,17]; reducing corporate innovation ability [18]; etc. This paper takes RTFEP as an entry point, explores the negative impact of EPU on RTFEP, and also shows that EPU will affect regional economic growth patterns.

The third is to provide representative empirical evidence for energy productivity governance in developing countries around the world. The empirical study in this paper is on the issue of energy productivity in China. As the largest developing country, China’s economic empirical evidence is of great significance to other countries, especially developing countries that need to deal with resource consumption and economic development mode transformation.

The remainder of this paper is organized as follows. Section 2 presents a theoretical analysis of the mechanism of EPU affecting RTFEP and puts forward research hypotheses. Section 3 describes the research design, variable structure, and data sources. Section 4 is the empirical estimation of the impact effect. In Section 5, the mechanism is tested. Section 6 is the heterogeneity analysis. Section 7 is the conclusion and discussion.

## 2. Theoretical Mechanism Analysis and Research Hypothesis

The market and the government are the indispensable “two hands” in the modern market economic system. They play an important role in the allocation of resources and macroeconomic regulation and are fundamental to achieve the transformation of the economic growth mode. This paper analyzes how the EPU affects RTFEP, focusing on two aspects: The price regulation of the energy market and local government intervention.

### 2.1. Energy Market Adjustment Mechanism

**Figure 1:**
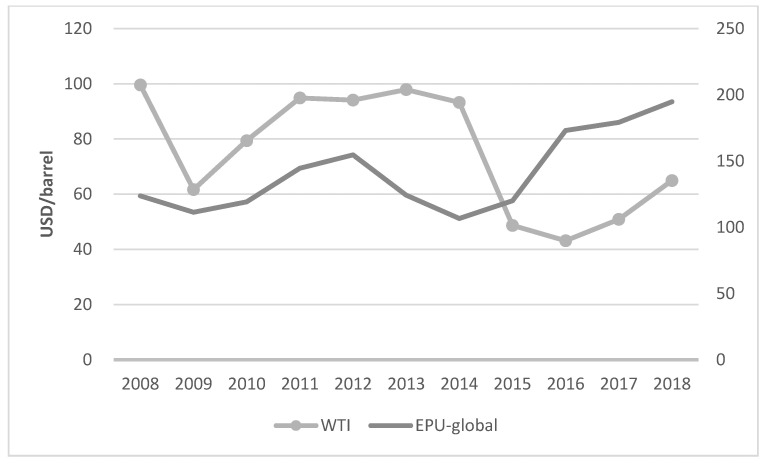
Trend of international crude oil prices (the left axis) and EPU-global (the right axis) from 2008 to 2018. The data are from the WTI crude oil price published by the US Energy Information Administration and the global economic policy uncertainty index calculated by Becker et al.

Changes in international oil prices can reflect the uncertainty of economic policies from the side. As shown in Figure 1, according to the WTI crude oil price (unit: USD/barrel) published by the US Energy Information Administration (data source: https://www.eia.gov/dnav/pet/hist/LeafHandler.ashx?n=pet&s=rwtc&f=a) (accessed on 19 September 2020). and the global economic policy uncertainty index (EPU-global) calculated by Becker et al., it can be seen that from 2008 to 2018 the trend of international oil prices and EPU-global is basically consistent, that is, when the uncertainty of global economic policy increases, international oil prices also show a rising trend.

Due to the particularity of the system and the economic environment, the marketization degree of different types of energy in China varies greatly. Electricity prices are tightly controlled by the government. Among them, the on-grid electricity price is determined in two ways: cost plus and bidding price. The electricity price for transmission and distribution is determined according to the cost plus method, and the electricity price for sale is determined by the government. Since 1992, China has gradually liberalized the coal price and transitioned from a planned price to market-oriented price. In 2002, the price of thermal coal was completely liberalized. In 2004, the coal–electricity price linkage was implemented. In 2006, the price of thermal coal was significantly liberalized, and the coal price entered the stage of complete marketization. In 1998, China carried out a major reform of the pricing mechanism for crude oil and refined oil products, and the price of domestic crude oil was fully integrated into the international market.

Therefore, when the international crude oil price rises due to the impact of EPU, the domestic electricity price will be maintained at a relatively stable level due to the government’s control. The price of domestic crude oil will increase due to the linkage with the international market. Coal is China’s most important energy consumer product. According to the data of the *BP Statistical Review of World Energy*, 58% of China’s total energy consumption in 2018 comes from coal. In terms of demand theory, while the price of crude oil rises, the prices of other energy sources will become relatively cheap. As a substitute for crude oil, the demand for coal will gradually increase. Compared with crude oil, the calorific value and combustion efficiency of coal is much lower, so the increase in demand for coal will increase the energy consumption per unit of output value and bring more environmental pollution problems, which will reduce RTFEP. Zeng and Jin [8] conducted an empirical analysis of the relationship between energy productivity and energy structure. The study found that reducing the use of coal and increasing the use of oil, electricity, and natural gas can increase energy productivity.

To sum up, an increase in EPU will lead to a rise in energy prices, especially the price of oil, which will further increase the proportion of coal in the energy consumption structure and affect the improvement of RTFEP.

**Hypothesis 1:**  *EPU reduces the RTFEP by changing the energy consumption structure.*

### 2.2. Local Government Intervention Mechanism

#### 2.2.1. Economic Policy Uncertainty and Government Intervention

The turmoil in the international environment will affect the formulation and implementation of a country’s economic policies and will cause uncertainty of it. Economic policy uncertainty refers to the fact that economic entities cannot accurately predict whether, when, or how the government will change the current economic policy [19]. In the short term, the speed at which central and local government policies are issued will slow down, the policies will lack a clear direction, and the intensity of policies will also decrease. Real option theory [20] states that when there is an adjustment cost (irreversible investment), the investment opportunity can be regarded as an option held by the enterprise, and the rise in uncertainty can increase the marginal investment cost of enterprises by increasing the option value, thus inhibiting the investment of enterprises. In this case, the degree of government intervention in the economy is reduced, the market’s ability to allocate resources is gradually reduced, and the negative impact of uncertainty on the macroeconomy is further deepened, which has a huge impact on economic recession. Albagli [21] believes that the spread of uncertainty is the most prominent feature of economic depression.

Under the socialist market economy system, the government’s strengthening of macroeconomic regulation and control is an important means to make up for market deficiencies and ensure the stable operation of the economy. Keynes first proposed the theory of government intervention in the economy. He believed that supply cannot automatically create demand, that total social supply and total demand cannot automatically reach equilibrium, and that government can improve the situation of insufficient effective demand by expanding public consumption and public investment expenditures and implementing expansionary fiscal policies. Joseph Stiglitz analyzed the government intervention theory from two perspectives: market failure theory and government economic function theory. On the one hand, the main role of government intervention is to compensate for market failures through “visible hands”; on the other hand, the government’s own failures are controllable. Under appropriate policies, government intervention can bring pareto improvement and improve the production efficiency of factors. Therefore, in the long run, the government will issue clear economic stimulus policies and implement expansionary fiscal policies to mitigate the economic recession caused by uncertainty in the context of continued turbulence in the macroeconomic environment. Hirst [22] believes that the government’s mandatory policies can solve the defects and inefficiencies of resource allocation in the market.

#### 2.2.2. Government Intervention and Energy Productivity

There is no consistent conclusion about the effect of government intervention on energy productivity. Liu [23] takes resource-based cities as the research object and believes that the establishment of a powerful government intervention mechanism and improvement of the government’s ability to regulate the macroeconomy can help resource-based cities solve resource trap problems and improve the utilization efficiency of resources. Wang and Ji [24], using the SBM-Tobit two-stage model to measure the energy productivity of various provinces and cities, have found in their empirical studies that government intervention has a significant positive impact on energy productivity. Chen and Luo [25] believe that government intervention can reduce the negative externalities caused by energy consumption and thus improve the energy utilization efficiency. Pan and Luo [26], based on the theory of factor endowments, have found that government intervention has a significant effect on improving the “resource curse” caused by resource endowments.

Some scholars also believe that government intervention will have a negative impact on energy productivity. Wei and Shen [9] calculated provincial energy productivity based on the DEA model of constant return to scale and found that for every unit of increase in government fiscal expenditure, energy productivity would decrease by 0.46%. Qu and Yuan [27] studied the influencing factors of energy intensity in eastern and western regions of China and found that the greater the degree of government intervention, the lower the energy utilization efficiency. Lin and Du [28] believe that the distortion of the factor market caused by the initial allocation of government-led resources and price regulation inhibited the improvement of energy productivity. Shi and Shen [29] found through empirical analysis that industrial agglomeration under the background of government intervention would have a negative impact on energy productivity. Guo et al. [7] calculated inter-provincial total-factor energy productivity based on the SBM model, and empirical tests found that government intervention was negatively correlated with energy productivity.

#### 2.2.3. Economic Policy Uncertainty, Government Intervention, and Energy Productivity

The financial crisis of 2008 was a representative event that triggered economic policy uncertainty. Compared with 2007, the global economic policy uncertainty index in 2008 increased from 69.8 to 123.7, with an increase of 77.4% [3]. The China economic policy uncertainty index increased by 107.3% from 50.4 to 104.6 [3]. In November 2008, the Chinese government launched ten policies to boost domestic demand and economic growth, requiring a total of CNY 4 trillion to be invested by the end of 2010. The implementation of this policy has successfully reached the target of 8% GDP growth and achieved remarkable results in economic recovery and the improvement of people’s livelihood. However, with about 47% of the investment directed to large-scale engineering projects such as roads, railways, airports, and water and rural infrastructure, which are already suffering from overcapacity and inefficient energy use, the stimulus adds to the problem. At the same time, in terms of China’s national conditions, the existing official tournament system leads to the competition between local governments, mainly manifesting as the competition of economic growth with GDP as the core [30], while the competition of GDP mainly depends on the growth of investment scale According to statistics, local governments have invested nearly CNY 18 trillion in the stimulus package. Therefore, under the incentive of political promotion, energy consumption and environmental pollution caused by GDP competition also aggravate the problem of low energy productivity to some extent.

Based on the above analysis, this paper believes that under the background of uncertain economic policies, the government will exert the power of the “visible hand” to stimulate economic growth by expanding fiscal expenditure and smooth out economic fluctuations. However, a large amount of investment in infrastructure will increase the problem of overcapacity. At the same time, excessive competition under the official championship system will make the negative externalities of economic growth become increasingly apparent, which will affect the improvement of regional total-factor energy productivity.

**Hypothesis 2:**  *EPU reduces the RTFEP through government intervention mechanisms.*

**Figure 2:**
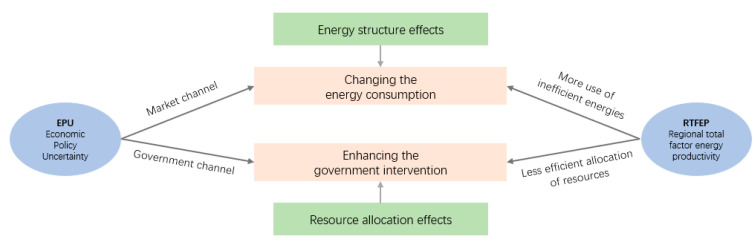
Theoretical framework.

Therefore, the following theoretical framework (Figure 2) of EPU affecting RTFEP was constructed.

## 3. Research Design and Data Sources

### 3.1. Research Design and Model Construction

This paper studies the impact of EPU on RTFEP, mainly including two aspects: one is to estimate the impact of EPU on RTFEP; the second is to verify the mechanism of the influence of EPU on RTFEP. Therefore, this section focuses on these two aspects in describing the research design.

(1) Effect estimation of EPU on RTFEP

EPU belongs to the economic index at the macrolevel, while RTFEP is at the regional level. Therefore, this paper chooses to build a prefecture-level econometric model to estimate the impact of EPU on RTFEP. The model is as follows:

(1) $$E_{it}=\alpha_{0\;}+\alpha_{1}\cdot U_{it}+\alpha_{2}\cdot X_{it}+year_{t}+\delta_{i}+\varepsilon_{it}$$

In Equation (1), the interpreted variable$\;E_{it\;}$is the total-factor energy productivity in year t of city i; the core explanatory variable $U_{it\;}$is the economic policy uncertainty in year t of city i; $X_{it}$ is the control variable; $year_{t}$ is the time trend effect (because the explanatory variables of economic policy uncertainty only change in time, time fixed effects are replaced by time trends to control the effects of unobservable factors that change over time); $\delta_{i}$ is the regional fixed effect; and $\varepsilon_{it}$ is the error term.

(2) Mechanism verification of EPU on RTFEP

This paper will verify the mechanism of EPU affecting RTFEP from the perspective of the energy market adjustment mechanism and government administrative intervention.

(a) Energy market regulation

EPU will have an impact on RTFEP by affecting energy prices and changing the structure of energy consumption.

(2) $$M_{it}=\beta_{0\;}+\beta_{1}\cdot U_{it}+\beta_{2}\cdot X_{it}+year_{t}+\delta_{i}+\varepsilon_{it}$$

In Equation (2), the interpreted variable$\;M_{it}\;$is the energy prices and energy consumption structure in year t of city i; the core explanatory variable $U_{it\;}$is the economic policy uncertainty in year t of city i; $X_{it}$ is the control variable; $year_{t}$ is the time trend effect; $\delta_{i}$ is the regional fixed effect; and $\varepsilon_{it}$ is the error term.

(b) Government economic intervention

Facing the macroeconomic recession accompanied by economic policy uncertainties, the government will stimulate economic growth through the introduction of expansionary fiscal policies. The negative externalities of the policies will have an impact on the region’s overall energy productivity.

(3) $$G_{it}=\lambda_{0\;}+\lambda_{1}\cdot U_{it}+\lambda_{2}\cdot X_{it}+year_{t}+\delta_{i}+\varepsilon_{it}$$

In Equation (3), the interpreted variable$\;G_{it}\;$is the government intervention in year t of city i; the core explanatory variable $U_{it\;}$is the economic policy uncertainty in year t of city i; $X_{it}$ is the control variable; $year_{t}$ is the time trend effect; $\delta_{i}$ is the regional fixed effect; and $\varepsilon_{it}$ is the error term.

### 3.2. Variable Measurement and Data Sources

(1) Core explanatory variables

**Figure 3:**
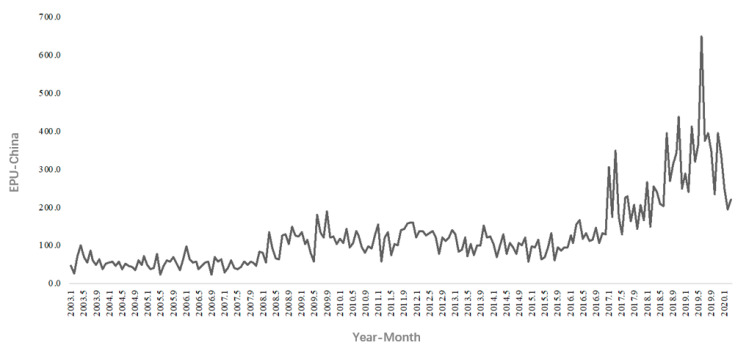
Monthly index of EPU in China from 2003 to 2020.

The core explanatory variable of this paper is economic policy uncertainty. Baker, Bloom, Davis, and Wang constructed the China economic policy uncertainty index in 2013 (the China economic policy uncertainty index compiled by Baker et al. was completed in 2013 and published in the 4th *Quarterly Journal of Economics* in 2016) [3,31,32]. The index is based on the Hong Kong English newspaper *South China Morning Post*. The frequency of articles about uncertainties in China’s economic policy eventually resulted in a monthly index of uncertainty in China’s economy. On this basis, Davis et al. took the *People’s Daily* and *Guangming Daily* as the analysis objects and developed the China economic policy uncertainty index based on mainland newspapers (data source: http://policyuncertainty.com/china_epu.html) (accessed on 1 December 2020). Since the research object of this paper is the prefecture-level cities in China’s interior, the economic policy uncertainty index based on mainland newspapers that better reflects the development of the central government’s economic policies was selected and was divided by 100 as the explained variable in this article. As shown in Figure 3, the China economic policy uncertainty index has shown an overall upward trend from 2003 to 2017. Specifically, EPU increased slightly from 2008 to 2016 and increased significantly from 2017 to early 2020. Therefore, it is necessary to study the impact of economic policy uncertainty on regional economic development.

From the measurement method of economic policy uncertainty, it can be seen that economic policy uncertainty is a macroindicator at the national level, reflecting the fluctuation and stability of the whole country’s economic policy. It can be considered that the macroeconomic uncertainty of a country’s economic policy is exogenous to the energy productivity of specific areas within the country. Therefore, choosing the economic policy uncertainty index of Baker et al. can better avoid the possible endogenous problem in the estimation. This indicator of economic policy uncertainty is also widely used to analyze the impact on the regional economy [13], corporate cash holdings [33], and the economic cycle [34].

(2) Explained variables

The explained variable in this paper is the regional total-factor energy productivity. The existing research has various methods for measuring energy productivity. According to the factors of input and output, they can be divided into single-factor indicators and total-factor indicators. At the same time, different measurement methods also reflect the differences in economic growth methods based on energy factors. The single-factor indicator is a partial-factor indicator reflecting the relationship between energy consumption and effective output in economic activities [35] and is usually measured by the energy consumption intensity per unit of GDP. Shi [36] used the reciprocal of energy consumption intensity to measure energy productivity and analyzed the regional differences in energy productivity and energy-saving potential in China. The calculation of single-factor indicators is simple, and it is also convenient for country comparison, but it cannot reflect the substitution effect of production factors such as labor and capital on energy, resulting in the problem of exaggerated energy productivity [37]. Yang and Shi [38] compared single-factor and total-factor energy efficiency indicators and found that the energy efficiency indicators measured by different total-factor methods have stronger consistency, and at the same time, total-factor indicators can better reflect the input of factors in various regions so as to make a more scientific and reasonable explanation of the utilization of energy productivity. From the perspective of economic growth, the accumulation of factors and the improvement of productivity are the two main sources of economic growth [39]. Economic growth is generally divided into two types: one is the growth achieved by increasing the input of natural resources, capital, and labor, and the other is achieved by improving the production efficiency [40]. Therefore, the way of measuring energy productivity by taking energy as a single input factor essentially reflects the extensive economic growth mode driven mainly by factor input.

Therefore, in order to make up for the shortcomings of the single-factor index, more and more scholars have begun to use the total-factor index for measurement. Hu and Wang [37] pioneered the use of data envelopment analysis (DEA) to measure the total-factor energy productivity of 29 provinces and municipalities in China. Among them, the input variable is labor, capital, energy consumption, and biomass energy, and the output variable is GDP. Subsequently, Zeng et al. [8,41], and Li and Huo [42] also used DEA to measure and analyze China’s energy productivity. The calculation method of the total-factor index considers that the increase in energy productivity comes from the productivity contribution of multiple input factors rather than the contribution of the accumulation of single input factors. This reflects the direction of economic growth towards intensive change to some extent.

However, although this method incorporates labor and capital factors into the DEA model, it does not take into account the impact of undesirable output from energy consumption on energy productivity. Later, Yuan et al. [43], Wang et al. [44], Yeh et al. [45], and Xu and Guan [46] used pollutants as an input, converted it into a desired output by taking the reciprocal or multiplying by “−1”, and calculated the total-factor energy productivity based on the Shephard distance function. However, this kind of treatment of pollutants is contrary to the actual production process, and it also will damage the convexity requirements of the model [35]. In order to solve this problem, Tone [47] incorporated slack variables into the objective function and constructed a non-radial and non-angle SBM model. This model not only solves the non-efficiency problem caused by input slack but also avoids deviations caused by radial and angular selection. Moreover, Tone [48] has further improved the model, incorporated undesirable output (environmental pollution) into the evaluation system, and comprehensively measured energy productivity from multiple inputs and multiple outputs and angles. The way of incorporating pollutants into the energy productivity calculation model is based on intensive economic growth, which further considers the importance of green development for economic growth and is an important performance of sustainable economic growth.

This paper believes that energy productivity is an important representation of the regional economic growth mode. In the context of current high-quality economic development, it is necessary to consider the total-factor energy productivity under the input of multiple factors and the negative impact of undesirable output on economic growth. In this paper, the DEA-SBM method taking environmental pollution into account will be used as the main measure of regional energy productivity, and the differences between the single-factor and traditional DEA approach will also be compared.

This paper uses three methods to measure the energy productivity of 281 prefecture-level cities (due to the lack of data, this article eliminated 12 out of 293 prefecture-level cities in China, including: Chaohu City in Anhui Province, Sansha City and Danzhou City in Hainan Province, Haidong City in Qinghai Province, and Turpan City and Hami City in Xinjiang Uygur Autonomous Region and Tibet Autonomous Region (Lhasa, Shigatse, Changdu, Nyingchi, Shannan, Naqu)) and 4 municipalities in China from 2003 to 2017.

(a) Single-factor energy productivity

The indicator uses energy consumption as an input variable and GDP as the output. It is measured by GDP divided by energy consumption (i.e., the reciprocal of energy intensity). In order to facilitate comparison with the other two methods, the results were normalized (divided by the maximum value). The energy consumption is the annual electricity consumption (10,000 kw/h); liquefied petroleum gas (ton) and gas (10,000 m^3^) were converted into the unit of 10,000 tons of standard coal, and then we summed them and took the logarithm. In order to eliminate the influence of price factors, the consumer price index (CPI) based on 2003 was used to deflate GDP and take the logarithm. The data of energy consumption and GDP come from the *China City Statistical Yearbook*, and the CPI comes from the National Bureau of Statistics of China.

(b) Total-factor energy productivity based on DEA–Malmquist model

DEA is a non-parametric test method developed on the basis of the concept of relative efficiency evaluation. It can be divided into three types: The CCR model, BCC model, and DEA–Malmquist model. The first two types are constant returns to scale and variable returns to scale which belong to the traditional DEA model and can only horizontally compare the production efficiency of DMUs at the same time point. The DEA–Malmquist index model can measure the dynamic changes in the production efficiency of DMUs in different periods, so we chose the DEA–Malmquist index model to measure the total-factor energy productivity.

The explained variable in this paper:

(4) $$\rho^{*}=min\frac{1-\frac{1}{m}\sum_{i=1}^{m}\frac{s_{i}^{-}}{x_{i0}}}{1+\frac{1}{s_{1}+s_{2}}\left(\;\sum_{r=1}^{s_{1}}\frac{s_{r}^{g}}{y_{r_{o}}^{g}}+\sum_{r=1}^{s_{2}}\frac{s_{r}^{b}}{y_{r_{0}}^{b}}\;\right)}\;s.t.\;x_{o}=X\lambda +s^{-}\;y_{0}^{g}=Y^{g}\lambda -s^{g}\;y_{0}^{b}=Y^{b}\lambda +s^{b}\;s^{-}\geq 0,\;s^{g}\geq 0,\;s^{b}\geq 0,\;\lambda \geq 0$$

In Equation (4), $x_{o}$, $y_{0}^{g}$, $y_{0}^{b}$ represent input, expected output, and undesired output elements; $\rho^{*}$ is the objective function; λ represents the input vector of the weight of the input elements; $s^{-}$, $s^{g},\;s^{b}$ represent the slack vectors of each input, expected output, and undesired output; m, $s_{1}$, $s_{2}$ represent the number of inputs, expected output, and undesired output, respectively. The more slack in the constraints on input and output, the lower the efficiency value of DMUs; when the slack vectors are all 0, the efficiency value $\rho^{*}$ of DMUs equals to 1, which means that the DMUs are valid, otherwise they are invalid and the input and output need to be improved to achieve optimal efficiency.

The three energy productivity measurement methods (Table 1) in this paper are summarized as follows.

### 3.3. Control Variables

EPU is a macroindicator, and it is difficult for factors at the microlevel to affect it. Therefore, this paper chooses from the influencing factors of RTFEP as the control variables. Based on the existing literature, we selected technological progress [5], environmental regulation [49], industrial structure [24], openness [50], and urbanization leve [51] as control variables.

Among them, the technological progress (TP) was measured by the internal expenditure of R&D funds. The provincial data were converted into prefecture-level city data according to the proportion of GDP of various cities and came from the National Bureau of Statistics of China. The environmental regulation (ER) was measured by the number of urban environmental protection laws and regulations, which was from China’s authoritative Wolters Kluwer legal database (data website: https://law.wkinfo.com.cn) (accessed on 22 November 2020). Industrial structure (IS) variables are expressed by the ratio of the output value of the secondary industry and the tertiary industry; the openness was measured by foreign direct investment in the city. The variables of the urbanization were measured by the proportion of non-agricultural population in the urban population. The data of industrial structure, openness, and urbanization were all derived from the *China City Statistical Yearbook*, and the control variables were all processed logarithmically.

The summary statistics of the above main variables are shown in Table 2.

## 4. Effect Estimation

(1) Benchmark regression results

This paper estimates the effect of EPU on RTFEP by using the fixed-effect model of Equation (1). The results are shown in Table 3. All of the three measurement methods show that EPU and RTFEP are significantly negatively correlated. Under the measurement of the single-factor and DEA–Malmquist indicator, for every unit of increase in EPU, the regional energy productivity will fall by 3% and 1%, respectively. According to the DEA-SBM, for every unit increase in EPU, RTFEP will decline by 5.6%. The possible reason is that the single-factor indicator is a manifestation of extensive economic growth. The calculation only considers the impact of energy as a single factor input on energy productivity but does not consider the role of labor and capital input. Although DEA–Malmquist measures energy productivity from the perspective of production efficiency under the intensive economic growth mode, the negative impact of pollutants is not taken into account. Therefore, the energy productivity indicators measured by these two methods are overestimated. In contrast, the method of incorporating pollutants as undesirable outputs into the SBM model is more scientific and reasonable, and the results are closer to green energy productivity in the context of sustainable development. Therefore, in the subsequent analysis, DEA-SBM is used to measure RTFEP.

It can be seen from model (6) that there is a significant positive correlation between technological progress and RTFEP. With the increase in regional R&D expenditure, technological innovation accelerates, which can improve the utilization of resources and promote the RTFEP. There is a significant positive correlation between environmental regulations and RTFEP. According to the Porter Hypothesis, proper environmental regulation can have an incentive effect on technological innovation to make up for the cost of environmental investment. When the benefits of innovation are greater than the costs paid for environmental regulation, energy productivity will be improved. The relationship between industrial structure and RTFEP is negative. Denison [52] and Maddison [53] first used the “structural dividend hypothesis” to explain the impact of industrial structure changes on energy productivity. The study claims that there are general differences in the productivity levels and growth rates of various sectors. When energy factors are allocated across sectors, the overall energy productivity will be improved. Since the secondary industry consumes much more energy than the tertiary industry, the increase in the proportion of the secondary industry has an inhibitory effect on energy productivity. Openness has a significant positive correlation with RTFEP. The higher the foreign direct investment, the higher the degree of openness and the greater the global competition that companies face. To some extent, this will encourage companies to improve their management models and increase energy efficiency. This result is consistent with Li et al. [49]. Urbanization is negatively correlated with RTFEP, but it is not significant. The possible reason is that, on the one hand, urbanization represents the improvement of the regional economic development, which can promote the development of technology and improve the efficiency of energy use; on the other hand, according to the scale effect of economic growth proposed by Grossman and Kureger [54] economic growth will increase the investment in resources, and it will also increase pollutant emissions, thus reducing regional energy productivity. Therefore, under the positive and negative effects, the urbanization level has no significant influence on RTFEP.

(2) Inspection of different samples and regions

a. Different samples

Considering the extreme value problem that may be caused by the difference in city levels, this paper selects different samples for regression to control the selective deviation. As shown in Table 4, model (1) represents the sample that excludes municipalities directly under the central government, model (2) is the sample that excludes cities at the sub-provincial level and above, and model (3) excludes cities at the provincial capital and above. The regression results show that after gradual elimination of the sample, the regression coefficients of EPU and control variables are still significant and relatively stable, which is consistent with the benchmark regression results.

b. Different regions

Further, this paper divides the sample into four regions: eastern, central, western, and northeastern, as well as the Yangtze River Delta, the Pearl River Delta, Beijing–Tianjin–Hebei, the Yangtze River Economic Belt, and the city clusters along the Belt and Road (as shown in Table 4 and Table 5). It can be seen from the grouped regression of different samples that EPU and RTFEP have a significant negative correlation, which is also consistent with the benchmark regression results.

## 5. Mechanism Verification

For a long time, China’s economic growth has been dependent on the growth of factor inputs. “High energy consumption, high pollution, and low efficiency” are typical characteristics of this extensive growth mode. At the same time, this type of economic growth driven by factor inputs has spawned unique government interventions, which have had an important impact on regional economic growth and energy productivity. Based on the analysis of the theoretical mechanism in the second part, we empirically tested the two mechanisms of energy market adjustment and local government intervention. Drawing on the existing literature [24,25,55], the energy price mentioned in Equation (2) was measured by the purchase price index of industrial producers (including raw material, fuel, and the power purchase price); the energy consumption structure was measured by the proportion of liquefied petroleum gas in energy consumption; and the government intervention variable involved in Equation (3) was measured by the proportion of local fiscal expenditure in GDP. The above data are all from the *China City Statistical Yearbook*.

(1) Energy market adjustment mechanism

The regression results according to Equation (2) are shown in Table 6 and mainly show the relationship between energy prices, EPU, and the energy consumption structure. It can be noticed from models (1) and (2) that energy prices have a positive relationship with EPU and a negative correlation with energy consumption structures. Meanwhile, model (3) shows that EPU and energy consumption structure are negatively correlated. Specifically, for every one-unit increase in EPU, energy price rises by 9.3%, and for every unit of energy price increase, the energy consumption structure drops by 1.5%, that is, the proportion of liquefied petroleum gas consumption in total energy consumption decreases. In addition, for every one-unit increase in EPU, the energy consumption structure drops by 0.9%. Model (4) incorporates energy productivity, energy consumption structure, and EPU into the model at the same time. The regression results show that there is a significant negative correlation between EPU and energy productivity, and for every one-unit increase in EPU, the energy productivity will drop by 5.7%, which is consistent with the benchmark regression results. At the same time, there is a significant positive correlation between the energy consumption structure and energy productivity. For every one-unit increase in the proportion of liquefied petroleum gas, RTFEP will rise by 10%. To sum up, the increase in EPU will bring about an increase in energy prices, especially the rise in oil prices, so the demand for oil will decline. Coal and oil are substitutes for each other. As a major energy consumer of coal, China’s rising oil price will lead to increased demand for coal, which is a highly energy-consuming resource. Therefore, EPU can reduce RTFEP through the adjustment of energy market prices and the consumption structure. Mechanism hypothesis 1 is verified.

(2) Government intervention mechanism

The regression results according to Equation (3) are shown in Table 7. Model (1) shows a significant negative correlation between EPU and RTFEP, which is consistent with the benchmark regression results. Model (2) shows a significant positive relationship between EPU and government intervention. For every unit of EPU, government fiscal expenditure will rise by 0.6%. Model (3) incorporates energy productivity, government intervention, and EPU into the model at the same time. Government intervention is significantly negatively correlated with RTFEP. For every unit increase in government fiscal expenditure, RTFEP will decrease by 32.6%. In short, the increase in EPU will cause economic recession, and the government will stimulate economic growth by increasing fiscal expenditure. According to the previous fiscal policy of the Chinese government, such as the “Four Trillion” stimulus plan issued during the financial crisis, a considerable portion of the capital flows into infrastructure construction industries, such as steel and cement. Such industries already have serious overcapacity problems, so the government’s intervention will make that problem more prominent. In addition, the promotion of the championship system among local government officials with economic growth as the core has led to a significant decline in RTFEP. Mechanism hypothesis 2 is verified.

## 6. Heterogeneity Analysis

The endowment and utilization of regional resources are closely related to the RTFEP. Therefore, this paper takes resource-based cities as an entry point to further explore the impact of EPU on the total-factor energy productivity of resource-based and non-resource-based cities, as well as different dominant resource cities. The resource-based cities were divided according to the “National Sustainable Development Plan for Resource-Based Cities in China (2013–2020)” issued by the State Council, and cities with different dominant resources were divided according to Zhang [56]. The results are shown in Table 8 and Table 9.

(1) Resource-based cities at different stages of development

In Table 8, it can be seen from models (1) and (2) that EPU will cause a significant negative impact on the total-factor energy productivity of resource-based and non-resource-based cities. For every unit increase, the total-factor energy productivity of these two types of cities will fall by 5.3% and 6%, respectively. Further, we considered whether there is heterogeneity between different types of resource-based cities. The regression results show that EPU has no significant impact on the energy productivity of growing cities but will have a significant negative impact on the energy productivity of mature, declining, and renewable cities. The possible reason may be related to the energy productivity of cities at different stages of development. Growing cities are in the rising stage of resource development, with great potential for resource security. The exploitation of resources can provide strong support for economic development, and the utilization efficiency of energy is at a relatively high level. Therefore, they are less impacted by the uncertainty of economic policies. Compared with the growing cities, the resource extraction process of mature cities is faster, and the mining intensity is greater. Therefore, they will face the problems of mining difficulty and rising mining costs. The sustainability of economic growth will decline, and the efficiency of energy use will also decrease. Under the impact of EPU, energy productivity will decline to a greater extent. Recessive cities have entered the end of resource extraction, facing the problems of resource depletion and increased pressure on the ecological environment, the lack of economic growth momentum, the unsustainability problem being increasingly prominent, and the efficiency of energy use being at a low level, so it will be greatly impacted by EPU. Although renewable cities have basically eliminated the dependence on resources, the development of strategic emerging industries is still in its infancy, and it is in the transition period of the economic structure. The utilization technology of energy is not yet mature, and the energy productivity has not yet reached a high level, so it is likely to be impacted.

(2) Resource-based cities with different dominant resources

Referring to Zhang. [56], resource-based cities are divided into oil and gas, coal, ferrous, non-ferrous, non-metallic, forest industry, and comprehensive categories—which refers to two or more types of dominant resources—according to different dominant resources. In Table 9, EPU has no significant impact on the energy productivity of oil and gas and forestry cities but has a significant negative impact on the remaining dominant resource cities. 

It may be that, compared with coal and ferrous metals, the energy productivity of oil and gas is relatively high, so the energy productivity of it is less impacted by EPU. Forest industry cities are dominated by vegetation cover and ecological protection, with little damage to the natural environment. The pollutant emissions brought by urban development are much lower than those of other cities, which provides sufficient impetus for the sustainable development of the economy. Therefore, the utilization efficiency of energy is very high, and it is difficult for the impact of EPU to affect it. In the process of energy utilization, ferrous, coal, non-metallic, and non-ferrous cities will produce a large number of pollutant emissions and serious environmental pollution, which is contrary to the concept of sustainable development. Therefore, energy productivity is lower and the impact of EPU is relatively large.

## 7. Conclusions

In the context of increasing uncertainty in global economic policies, how to change the economic growth mode to deal with the impact of uncertainty is a problem that all governments need to consider deeply. As an essential input factor in the process of economic growth, energy can promote the transformation of economic growth toward an intensive and sustainable direction by improving the efficiency of energy use and reducing the impact of EPU. This was the starting point of this study. Based on the China economic policy uncertainty index constructed by Baker et al. (2013) [31,57,58], we explored the effect, mechanism, and heterogeneity of EPU on RTFEP.

In terms of impact effects, this paper uses the panel fixed-effect model to estimate the impact of EPU on RTFEP from the prefecture city level. The regression results show that the single-factor method and the method without undesirable output will overestimate the actual energy productivity. In contrast, the SBM model incorporating undesirable output reflects sustainable economic growth and can reflect the RTFEP more scientifically and comprehensively. According to the calculation results of the SBM model, EPU has a significant negative correlation with energy productivity. For every one-unit increase in EPU, RTFEP will decrease by 5.6%. At the same time, the regression results of the control variables show that technological progress, environmental regulation, and openness can promote the RTFEP. The industrial structure is inversely related to the RTFEP, and the urbanization will not have a significant impact on energy productivity. The above results are basically stable after gradually excluding samples and grouping according to administrative regions and urban agglomerations.

In terms of mechanism verification, this paper examines the ways in which EPU affects the RTFEP from the perspective of energy market adjustment and government intervention. First, EPU will reduce RTFEP through the “energy price-energy consumption structure” approach. Under the background of economic globalization, domestic EPU will be affected by international economic fluctuations. The rise in international oil prices is a manifestation of the rise in global EPU. For China, electricity prices are strictly controlled by the government, coal prices have been marketized, and domestic oil prices are interlinked with the international market. Therefore, the rise in international oil prices will first cause domestic oil prices to rise. As an alternative to oil, the demand for coal will increase, and the energy productivity of coal is much lower than that of oil, resulting in the decline of RTFEP. Second, rising EPU will reduce RTFEP through the “recession-government intervention” approach. When the uncertainty of economic policies increases, the speed of the government to introduce economic policies will slow down and the directivity will also decline. In order to avoid the risks brought by the uncertainty, enterprises as the main body of the market economy will reduce their investment willingness, and the overall economic situation will therefore enter a state of recession. Under the socialist market economic system, the government will strengthen intervention in the economy through positive fiscal policy. However, the implementation of these policies will inevitably increase the investment in the infrastructure industries, such as steel and cement, thereby exacerbating the industry’s overcapacity problem; the problem will become more prominent in the context of the local officials’ promotion tournament, which will eventually lead to a decline in regional energy productivity.

According to the heterogeneity analysis, resource-based cities are divided according to different development periods and different dominant types. Empirical regression finds that EPU will not have a significant impact on the energy productivity of growth, oil and gas, and forest industry cities, mainly because these cities have higher energy efficiency, and the negative impact of economic development on the ecological environment is smaller. In comparison, due to excessive resource exploitation and environmental pollution problems, the energy efficiency of mature, declining, non-ferrous, and black cities is lower, so the degree of impact of EPU is even more obvious.

## 8. Discussion and Limitations

Based on the above analysis, the following three policy recommendations are presented:

Optimize energy consumption structure. China is a large energy country dominated by coal consumption. Therefore, when the price of oil rises, the replacement cost of coal to oil is relatively low, which will result in a decline in energy efficiency. In the process of economic development, the region should focus on optimizing the energy consumption structure, reducing its dependence on coal and oil, transforming the extensive economic growth mode that comes at the cost of resource consumption, increasing R&D investment and technological innovation, stepping up the development of new and renewable sources of energy, and increasing the share of clean energy in the energy consumption structure. In the face of oil price shocks caused by EPU, on the one hand, energy utilization efficiency can be improved by upgrading energy utilization technology; on the other hand, it is possible to choose clean energy as an alternative to reduce the environmental damage caused by coal combustion, so as to improve total-factor energy productivity.

Regulate the field of government investment. At present, the Chinese government’s financial investment is mainly used for economic construction, but investment in social security, medical care, education, and other fields is relatively insufficient. Excessive investment in infrastructure projects will further aggravate the problem of overcapacity, which in turn will cause a decline in energy productivity. Therefore, when faced with economic recession caused by EPU, government intervention in the economy should be inclined to increase social supply and livelihood security and improve the public service system, appropriately reduce fiscal investment in industries with excess capacity, and promote fiscal expenditure shifts from “construction finance” to “public finance”, focusing more on the sustainability and quality of economic growth, rather than just increasing the growth rate, thus maximizing the positive role of the government in macroeconomic regulation.

Transformation of economic development. Excessive dependence on resources will make the energy productivity of resource-based cities face greater challenges under the impact of EPU. Overexploitation of resources and ecological damage will exacerbate the fragility of energy productivity. Therefore, the development process of resource-based cities should tend to promote the transformation of the economic development mode to the intensive mode and formulate differentiated economic development mode transformation strategies according to different stages and dominant types. For example, for mature cities, it is necessary to reasonably control the intensity of resource extraction and avoid the problem of resource depletion; for renewable cities, it is necessary to accelerate the layout of strategic emerging industries, help the industrial development of the city to achieve a smooth transition, and give play to the advantages of new industries and technologies to promote energy productivity.

However, the findings of this study have to be seen in the light of some limitations. This paper uses time series data and does not use full panel data due to limitations in access to data. In future studies, more complete data will be used to deepen our research. Secondly, the limited literature available for reference does not allow for a comprehensive comparison. On the other hand, this highlights the importance of this pioneering study. Furthermore, for the mechanism analysis, this paper examines the mechanism of EPU on RTFEP from the perspective of the market and the government. In future studies, the different perspectives will be further expanded, and more comprehensive mechanisms will be discussed to enrich the existing research findings. Finally, in the future, the authors will consider extending the relevant studies in this paper to other regions or countries to enrich the research in related fields.

## Figures and Tables

**Table 1:** Energy productivity measurement method.

	Single Factor	DEA	SBM	Economic Growth Mode Based on Energy Factors
Input	Energy consumption	Labor; capital; energy consumption	Labor; capital; energy consumption	Extensive growth
Output	GDP	GDP	GDP; sulfur dioxide; wastewater	Intensive growth
Method	GDP/energy consumption	DEA–Malmquist	DEA-SBM	Sustainable growth

**Table 2:** Summary statistics of the variables.

Variables	Obs	Mean	Std. Dev.	Min	Max
Single factor	4275	0.0703	0.0632	0.0004	1
DEA–Malmquist	4275	0.5061	0.0215	0.3009	1
DEA-SBM	4275	0.6794	0.1320	0.1929	1
EPU	4275	0.9929	0.4059	0.5044	2.0664
TP	4275	4.6324	1.4681	0.5301	11.9701
ER	4275	4.0728	0.8875	0	6.0521
IS	4275	0.8562	0.4331	0.0861	4.2655
Openness	4275	4.8520	1.9789	0	10.0995
Urbanization	4275	0.3914	0.2166	0.1245	0.9267

**Table 3:** Benchmark regression results.

	(1)	(2)	(3)	(4)	(5)	(6)
	Single Factor	Single Factor	DEA–Malmquist	DEA–Malmquist	DEA-SBM	DEA-SBM
EPU	−0.030 ***	−0.030 ***	−0.010 ***	−0.010 ***	−0.056 ***	−0.057 ***
	(0.00)	(0.00)	(0.00)	(0.00)	(0.00)	(0.00)
TP		0.028 ***		0.009 ***		0.069 ***
		(0.01)		(0.00)		(0.01)
ER		0.002		0.003 ***		0.025 ***
		(0.00)		(0.00)		(0.00)
IS		−0.022 ***		−0.010 **		−0.021 *
		(0.01)		(0.00)		(0.01)
Openness		0.003		−0.001		0.005 ***
		(0.00)		(0.00)		(0.00)
Urbanization		0.008		0.003		−0.006
		(0.02)		(0.01)		(0.02)
City fixed effect	YES	YES	YES	YES	YES	YES
Trend fixed	YES	YES	YES	YES	YES	YES
Constant term	−7.522 ***	3.430 *	2.566 ***	6.177 ***	−23.712 ***	7.288 **
	(0.45)	(1.98)	(0.24)	(1.01)	(0.48)	(3.18)
N	4275	4275	4275	4275	4275	4275
Adjusted R^2^	0.200	0.255	0.143	0.162	0.488	0.572
Section number	285	285	285	285	285	285

Notes: * Superscripts indicate significance at the 10% levels. ** Superscripts indicate significance at the 5% levels. *** Superscripts indicate significance at the 1% levels. Robustness standard errors of clustering to city level are in the parentheses.

**Table 4:** Inspection of different samples and regions.

	(1)	(2)	(3)	(4)	(5)	(6)	(7)
	Excluding Municipalities	Excluding Sub-Provincial Cities and Above	Excluding Provincial Capitals and Above	Eastern	Central	Western	Northeastern
EPU	−0.058 ***	−0.060 ***	−0.060 ***	−0.044 ***	−0.059 ***	−0.072 ***	−0.048 ***
	(0.00)	(0.00)	(0.00)	(0.00)	(0.00)	(0.01)	(0.01)
Control variables	YES	YES	YES	YES	YES	YES	YES
City fixed effect	YES	YES	YES	YES	YES	YES	YES
Trend fixed	YES	YES	YES	YES	YES	YES	YES
Constant term	7.709 **	6.921 **	6.025 *	19.243 ***	7.334	−0.815	2.460
	(3.19)	(3.27)	(3.18)	(6.59)	(9.33)	(6.52)	(2.14)
N	4215	3990	3750	1305	1200	1260	510
Adjusted R^2^	0.573	0.574	0.582	0.518	0.626	0.559	0.622
Section number	281	266	250	87	80	84	34

Notes: Control variables include technological progress, environmental regulation, industrial structure, openness, and urbanization. * Superscripts indicate significance at the 10% levels. ** Superscripts indicate significance at the 5% levels. *** Superscripts indicate significance at the 1% levels. Robustness standard errors of clustering to city level are in the parentheses.

**Table 5:** Inspection of different city clusters.

	(1)	(2)	(3)	(4)	(5)
	Yangtze River Delta	Pearl River Delta	Beijing–Tianjin–Hebei	Yangtze River Economic Belt	Belt and Road
EPU	−0.052 ***	−0.032 **	−0.035 ***	−0.060 ***	−0.069 ***
	(0.01)	(0.01)	(0.01)	(0.00)	(0.01)
Control variables	YES	YES	YES	YES	YES
City fixed effect	YES	YES	YES	YES	YES
Trend fixed	YES	YES	YES	YES	YES
Constant term	40.999 ***	39.098 **	56.920 **	4.727	−5.717
	(11.97)	(13.52)	(23.42)	(6.47)	(7.54)
N	615	135	195	1620	1065
Adjusted R^2^	0.664	0.458	0.477	0.650	0.546
Section number	41	9	13	108	71

Notes: Control variables include technological progress, environmental regulation, industrial structure, openness, and urbanization. ** Superscripts indicate significance at the 5% levels. *** Superscripts indicate significance at the 1% levels. Robustness standard errors of clustering to city level are in the parentheses.

**Table 6:** Energy market adjustment mechanism.

	(1)	(2)	(3)	(4)
EPU	0.093 ***		−0.009 ***	−0.057 ***
	(0.00)		(0.00)	(0.00)
Energy price		−0.017 **		
		(0.01)		
Energy consumption structure				0.100 **
				(0.05)
Control variables	YES	YES	YES	YES
City fixed effect	YES	YES	YES	YES
Trend fixed	YES	YES	YES	YES
Constant term	52.833 ***	4.005 *	2.557	7.031 **
	(7.30)	(2.18)	(2.13)	(3.18)
N	4275	4275	4275	4275
Adjusted R^2^	0.860	0.500	0.501	0.574
Section number	285	285	285	285

Notes: Control variables include technological progress, environmental regulation, industrial structure, openness, and urbanization. * Superscripts indicate significance at the 10% levels. ** Superscripts indicate significance at the 5% levels. *** Superscripts indicate significance at the 1% levels. Robustness standard errors of clustering to city level are in the parentheses.

**Table 7:** Government intervention mechanism.

.	(1)	(2)	(3)
EPU	−0.057 ***	0.006 ***	−0.056 ***
	(0.00)	(0.00)	(0.00)
Government intervention			−0.326 ***
			(0.09)
Control variables	YES	YES	YES
City fixed effect	YES	YES	YES
Trend fixed	YES	YES	YES
Constant term	7.288 **	−15.909 ***	2.100
	(3.18)	(3.51)	(3.34)
N	4275	4275	4275
Adjusted R^2^	0.572	0.810	0.579
Section number	285	285	285

Notes: Control variables include technological progress, environmental regulation, industrial structure, openness, and urbanization. ** Superscripts indicate significance at the 5% levels. *** Superscripts indicate significance at the 1% levels. Robustness standard errors of clustering to city level are in the parentheses.

**Table 8:** Resource-based city (different development stages).

	(1)	(2)	(3)	(4)	(5)	(6)
	Resource-Based	Non-Resource-Based	Growing	Mature	Recessive	Renewable
EPU	−0.053 ***	−0.060 ***	−0.027	−0.063 ***	−0.033 ***	−0.057 ***
	(0.01)	(0.00)	(0.02)	(0.01)	(0.01)	(0.01)
Control variables	YES	YES	YES	YES	YES	YES
City fixed effect	YES	YES	YES	YES	YES	YES
Trend fixed	YES	YES	YES	YES	YES	YES
Constant term	5.541	8.538 *	29.787	3.788	−1.381	22.324 *
	(4.02)	(4.80)	(25.54)	(6.82)	(3.44)	(10.89)
N	1695	2580	195	930	345	225
Adjusted R^2^	0.534	0.598	0.335	0.548	0.814	0.580
Section number	113	172	13	62	23	15

Notes: Control variables include technological progress, environmental regulation, industrial structure, openness, and urbanization. * Superscripts indicate significance at the 10% levels. *** Superscripts indicate significance at the 1% levels. Robustness standard errors of clustering to city level are in the parentheses.

**Table 9:** Resource-based city (different dominant resources).

	(1)	(2)	(3)	(4)	(5)	(6)	(7)
	Oil and Gas	Coal	Ferrous	Non-Ferrous	Non-Metallic	Forest Industry	Comprehensive
EPU	−0.029	−0.053 ***	−0.040 ***	−0.091 **	−0.069 ***	−0.061	−0.042 ***
	(0.02)	(0.01)	(0.01)	(0.03)	(0.02)	(0.03)	(0.01)
Control variables	YES	YES	YES	YES	YES	YES	YES
City fixed effect	YES	YES	YES	YES	YES	YES	YES
Trend fixed	YES	YES	YES	YES	YES	YES	YES
Constant term	−11.871	12.200 **	14.839	−34.417	5.476	22.324 *	22.324 *
	(12.67)	(5.80)	(11.50)	(19.66)	(13.10)	(10.89)	(10.89)
N	165	585	135	165	165	90	390
Adjusted R^2^	0.513	0.570	0.855	0.661	0.687	0.592	0.381
Section number	11	39	9	11	11	6	26

Notes: Control variables include technological progress, environmental regulation, industrial structure, openness, and urbanization. * Superscripts indicate significance at the 10% levels. ** Superscripts indicate significance at the 5% levels. *** Superscripts indicate significance at the 1% levels. Robustness standard errors of clustering to city level are in the parentheses.

## Data Availability

The data that has been used is confidential.
